# Supplementary material for: Sex differences in the effects of individual anxiety state on regional responses to negative emotional scenes
Source: Biol Sex Differ. 2024 Feb 13;15:15. doi: 10.1186/s13293-024-00591-6 (PMC10863151; doi:10.1186/s13293-024-00591-6)
Supplement: Supplementary file 1 — Additional file 1: Figure S1. Design matrix: Full factorial model with each subject's "Neg-Neu" contrast images, 'sex' as a two-level factor, STAI-Score × sex as a covariate, and age as a controlled covariate. Column 1: “Neg-Neu” in men, Column 2: “Neg-Neu” in women, Column 3: STAI-Score in men, Column 4: STAI-Score in women, Column 5: age. Figure S2. Brain activations of “Neg-Neu” contrast: one sample t test in (A) all, (B) men, and (C) women; voxel p<0.001, uncorrected. Color bars show voxel T values, with warm and cool color each for positive (Neg > Neu) and negative (Neu > Neg) activations. Table S1. Mediation model β and p-values (X/M/Y: independent/mediating/dependent variable) with ‘age’ as covariate. Figure S3. Regional parametric estimates (β; average activity) during “negative” and “neutral” picture condition in all subjects (men + women) (A), and in women (B–E). Note: the difference in activation during negative and neutral conditions is denoted with p-value (under the bar) in Wilcoxon signed-rank test. LG lingual gyrus, mPFC medial prefrontal cortex, R-SFG right superior frontal gyrus, L-SFG left SFG. [file 13293_2024_591_MOESM1_ESM.docx]

**Additional file 1**

**Chaudhary et al. Sex differences in the effects of individual anxiety state on regional responses to negative emotional scenes**

Shefali Chaudhary, Hak Kei Wong, Yu Chen, Sheng Zhang, Chiang-Shan R. Li


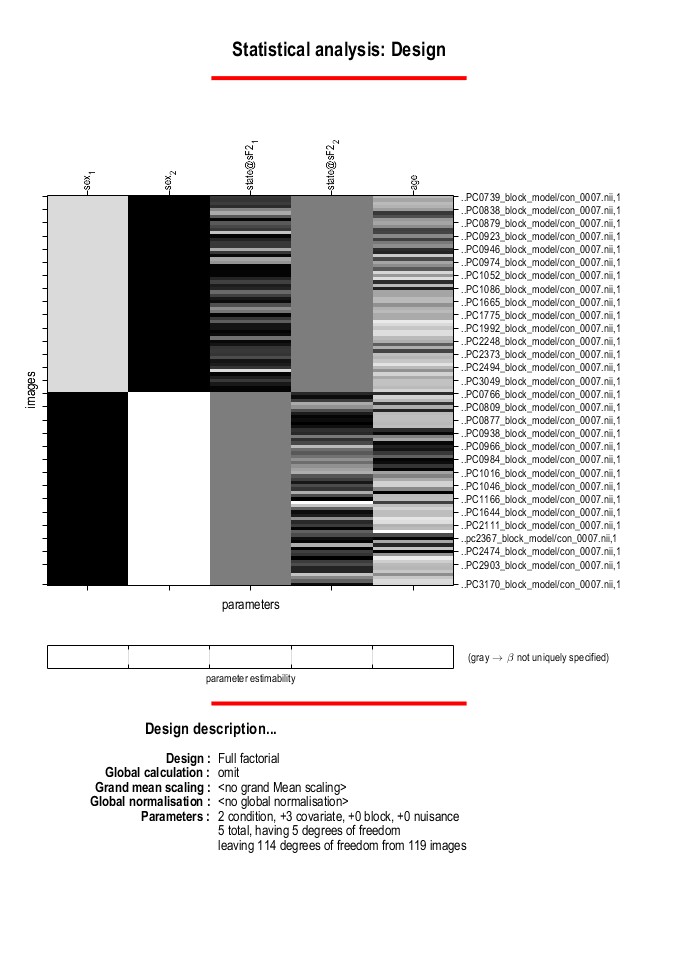


**Figure S1**. Design matrix: Full factorial model with each subject's "Neg-Neu" contrast images, 'sex' as a two-level factor, STAI-Score × sex as a covariate, and age as a controlled covariate. Column 1: “Neg-Neu” in men, Column 2: “Neg-Neu” in women, Column 3: STAI-Score in men, Column 4: STAI-Score in women, Column 5: age.

**Supplemental methods: mediation analysis**

***Mediation analysis***

To examine the interrelationships of BOLD responses (beta estimates) of identified regions, age, GP, and AUDIT score, we conducted mediation analyses using a single-mediator model. The methods were detailed in our previous work [1,2]. Briefly, in a mediation analysis, the relation between the independent variable X and dependent variable Y; that is, X → Y is tested to determine whether it is significantly mediated by a variable M. The mediation test is performed using the following three regression equations:

Y=i_1_+cX+e_1_

Y=i_2_+c′X+bM+e_2_

M=i_3_+aX+e_3_

where a represents X → M, b represents M → Y (controlling for X), c' represents X → Y (controlling for M), and c represents X → Y. The constants i_1_, i_2_, i_3_ are the intercepts, and e_1_, e_2_, e_3_ are the residual errors. In the literature, a, b, c, and c' are commonly referred to as “path coefficients,” Variable M is said to be a mediator of connection X → Y, if (c – c'), which is mathematically equivalent to the product of the paths a × b, is significantly different from zero. If (c – c') is different from zero and the paths a and b are significant, one concludes that X → Y is mediated by M. In addition, if path c' is not significant, it indicates that there is no direct connection from X to Y and that X → Y is completely mediated by M. The analysis was performed with package ‘Medsem’ in STATA [3]. To test the significance of the mediation effect, we used the bootstrapping method as it is generally considered advantageous to the Sobel test [4].


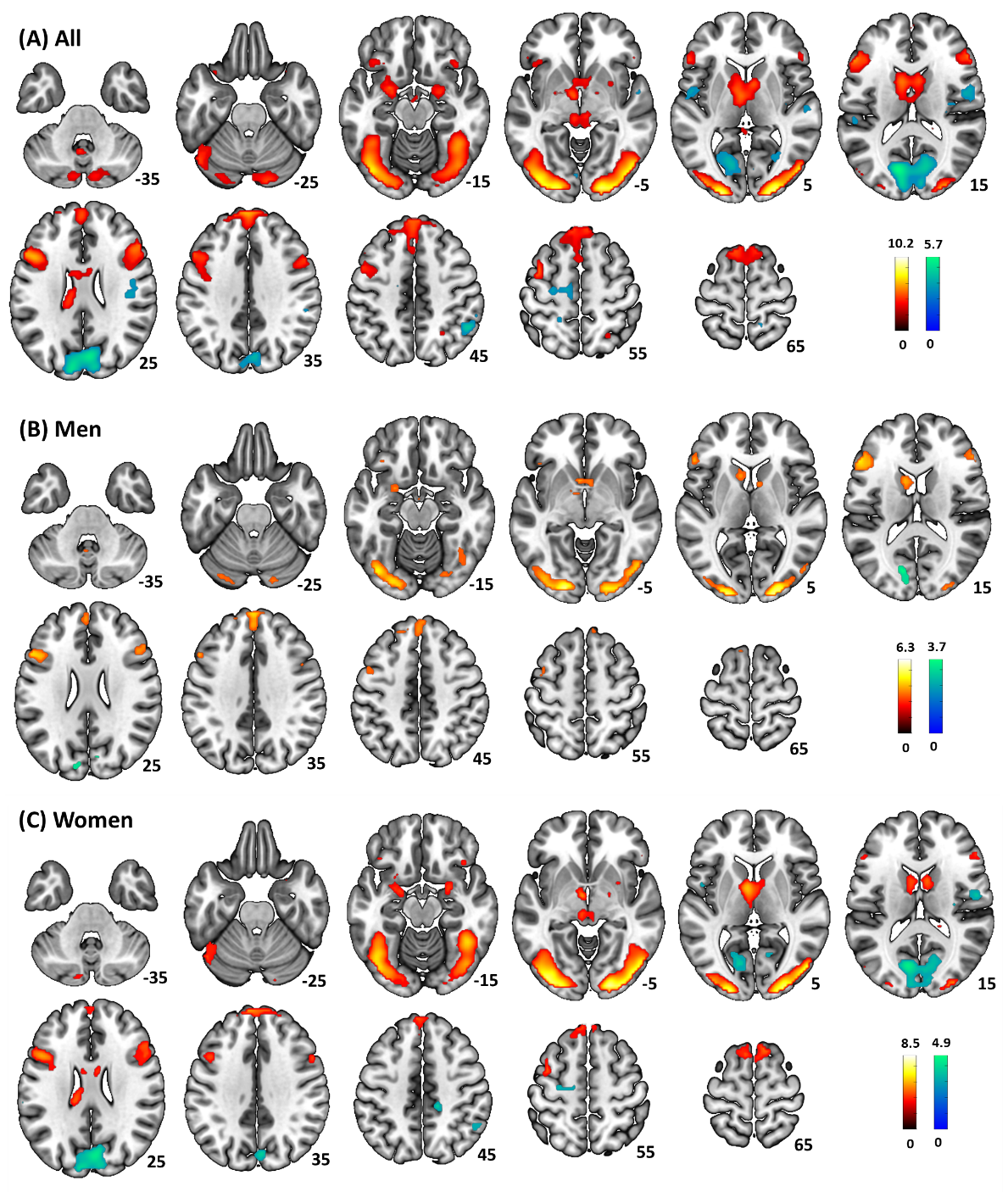


**Figure S2.** Brain activations of “Neg-Neu” contrast: one sample t test in (A) all, (B) men, and (C) women; voxel p<0.001, uncorrected. Color bars show voxel T values, with warm and cool color each for positive (Neg > Neu) and negative (Neu > Neg) activations.

**Table S1**. Mediation model β and p-values (X/M/Y: independent/mediating/dependent variable) with ‘age’ as covariate

| X | M | Y | β, p- value | | | | |
| --- | --- | --- | --- | --- | --- | --- | --- |
|  |  |  | X 🡪 M | M 🡪 Y | X 🡪 Y | Direct  X 🡪 Y | Indirect  X 🡪 Y |
| Men |  |  |  |  |  |  |  |
| State | mPFC β | RT | -0.003,  0.678 | 0.03,  0.597 | -0.002,  0.504 | -0.002,  0.504 | -0.0001,  0.744 |
| State | mPFC FC β | RT | 0.001,  0.190 | -0.06,  0.917 | -0.002,  0.504 | -0.002,  0.521 | -0.00006,  0.917 |
| Women |  |  |  |  |  |  |  |
| State | mPFC β | RT | -0.04,  <0.001 | -0.02,  0.435 | 0.01,  <0.001 | 0.009,  0.001 | 0.001,  0.445 |
| State | mPFC FC β | RT | 0.004,  <0.001 | 1.28,  0.001 | 0.01,  <0.001 | 0.005,  0.052 | 0.005,  0.006* |

‘*’ significant mediation effect


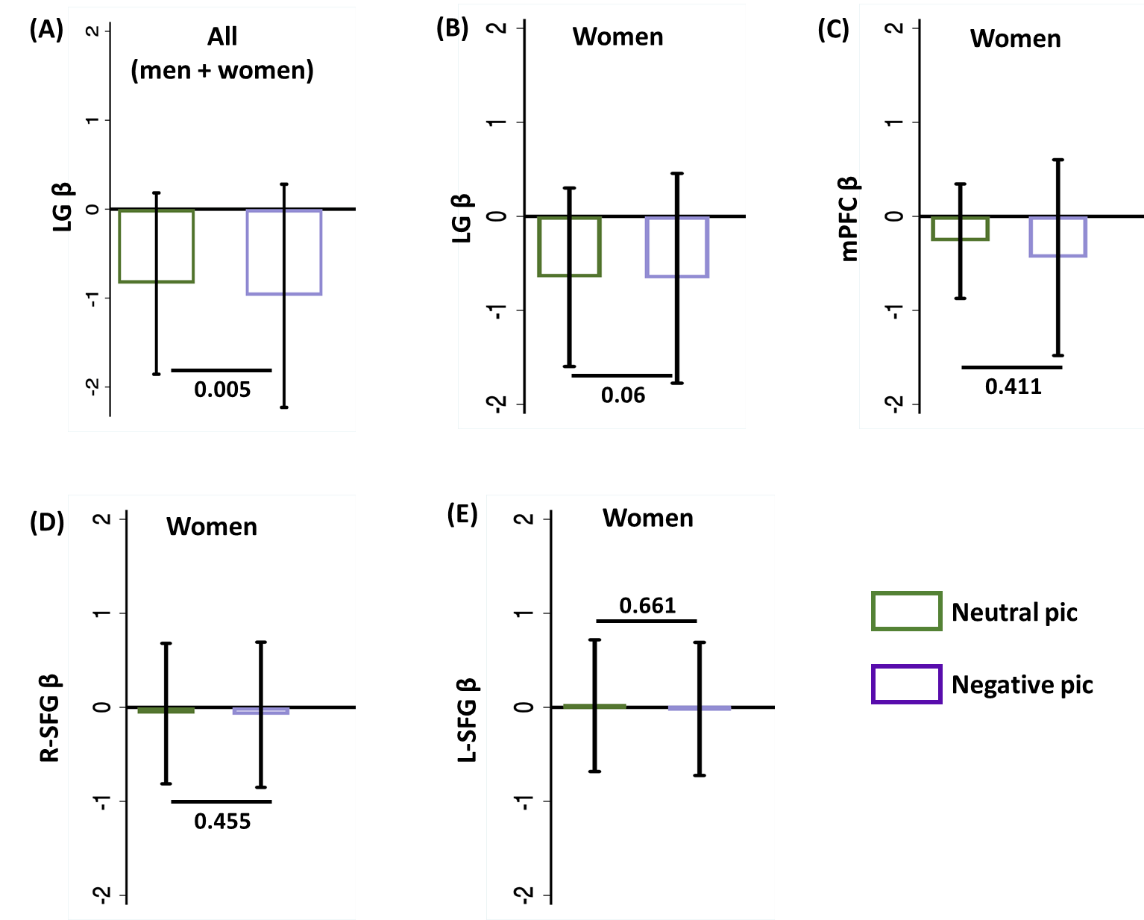


**Figure S3.** Regional parametric estimates (β; average activity) during “negative” and “neutral” picture condition in all subjects (men + women) (A), and in women (B-E). Note: the difference in activation during negative and neutral conditions is denoted with p-value (under the bar) in Wilcoxon signed-rank test. LG: lingual gyrus, mPFC: medial prefrontal cortex, R-SFG: right superior frontal gyrus, L-SFG: left SFG.

References:

1. Zhornitsky S, Zhang S, Ide JS, Chao HH, Wang W, Le TM, et al. Alcohol Expectancy and Cerebral Responses to Cue-Elicited Craving in Adult Nondependent Drinkers. Biol psychiatry Cogn Neurosci neuroimaging. 2019;4:493–504.

2. Wang W, Zhornitsky S, Le TM, Zhang S, Li C-SR. Heart Rate Variability, Cue-Evoked Ventromedial Prefrontal Cortical Response, and Problem Alcohol Use in Adult Drinkers. Biol psychiatry Cogn Neurosci neuroimaging. 2020;5:619–28.

3. Mehmetoglu M. medsem: a Stata package for statistical mediation analysis. Int J Comput Econ Econom. 2018;8:63.

4. Zhao X, Lynch J, Chen Q. Reconsidering Baron and Kenny: Myths and Truths About Mediation Analysis. J Consum Res. 2010;37:197–206.
